# Sequencing meets machine learning to fight emerging pathogens: A preview

**DOI:** 10.1016/j.patter.2022.100448

**Published:** 2022-02-11

**Authors:** Artur Yakimovich

**Affiliations:** 1 Center for Advanced Systems Understanding (CASUS), Helmholtz-Zentrum Dresden-Rossendorf e.V. (HZDR), Görlitz 02826, Germany; 2 Bladder Infection and Immunity Group (BIIG), Department of Renal Medicine, Division of Medicine, University College London, Royal Free Hospital Campus, London NW3 2PF, United Kingdom; 3 Artificial Intelligence for Life Sciences CIC, Dorset BH16 6FA, United Kingdom; 4 Roche Pharma International Informatics, Roche Diagnostics GmbH, Mannheim 68305, Germany

## Abstract

In searching for SARS-CoV variants-of-concern, pathogen sequencing is generating an impressive amount of data. However, beyond epidemiological use, these data contain cues fundamental to our understanding of pathogen evolution in the human population. Yet, to harness them, further development of computational methodology, such as machine learning, may be required. This preview discusses updates in machine learning to understand emerging pathogens.

## Main text

With over 300 million confirmed cases to date,^1^ SARS-CoV-2 demonstrates the sheer extent to which a pandemic pathogen can transform our interconnected world. However, unlike in many pandemics of the past, the availability of sequencing techniques^2^ has profoundly changed the amount of quasi-real-time information we have about the situation unfolding in front of us. The monumental sequencing effort undertaken by the scientific community has led to the accumulation of several million SARS-CoV-2 sequences already,^3^ offering an unprecedented research opportunity.

However, to tap into this opportunity, the biomedical community is in dire need of a qualitatively new set of tools. Indeed, picking up faint but pivotal patterns within several million SARS-CoV-2 sequences at high speed is an insurmountable task even for a large team of scientists. Instead, the new set of tools required for such tasks should be capable of seamlessly sifting through millions of sequences almost instantaneously. At the same time, these tools must be sensitive and specific enough to uncover yet unknown dependencies without generating false leads. These are exactly the capabilities machine learning (ML) has in store.^4^ While ML techniques were used in biology before,^5^ recent advances in computational algorithms and hardware have rekindled the popularity of ML within the biomedical field,^6^ particularly within infection biology (reviewed in Yakimovich, 2021^7^).

Adding to the ML toolset, Park and colleagues recently proposed a novel ML approach to identify discriminative genomic features in SARS-CoV-2 in a metaviromic fashion.^8^ In this approach, authors first collated a dataset consisting of 3,665 human and animal coronavirus genomes including SARS-CoV-2, MERS-CoV, and SARS-CoV. Furthermore, by comparing coronaviruses historically associated with higher case fatality rates combined with multiple sequence alignment and knowledge of genomic regions of interest, Park et al. have developed coronavirus pathogenicity (COPA) scores for every nucleotide in the SARS-CoV-2 genome. To achieve this, authors first compared a set of well-established ML algorithms including random forest, support vector machines, Bernoulli naive Bayes, gradient boosting, and multi-layer perceptron to determine their ability to identify genomes associated with pathogenic coronaviruses. Next, Park and colleagues integrated these approaches into a statistical metamodel allowing them to search for pathogenic hotspots within the SARS-CoV-2 genomes.

Employing this approach, authors generated 2,473 discriminative hotspots across the SARS-CoV-2 genome and compared them to the known genomic regions of interest. Remarkably, they demonstrate that the hotspots associated with SARS-CoV-2 spike (S) protein overlap with both the infamous furin cleavage site and the contact sites with angiotensin-converting enzyme 2 of the host cell. Another interesting hotspot Park et al. identified using their proposed approach corresponded with amino acid insertions allowing to differentiate betacoronaviruses from alpha- and gammacoronaviruses. Furthermore, authors decided to combine the knowledge of B and T immune-cell epitopes responsible for the immune response generation with the ML-generated hotspot map of the SARS-CoV-2 genome. They noticed that the high COPA pathogenic regions of the S and N proteins significantly overlapped with potential B cell epitopes. Finally, authors looked at the cross-correlation between COPA hotspots and the sequences of the known variants-of-concern. Among other observations, they noticed an overlap of several high-score residues and mutations for variant B.1.1.7, also known as the SARS-CoV-2 Alpha variant. Following these observations, Park and coworkers suggested potential usefulness of their methodology for guiding the design of future SARS-CoV-2 vaccines and epidemiological variant surveillance.

Together with the other efforts applying novel ML methodology to pathogen genomics,^9^^,^^10^ the work of Park et al. outlines the immense potential ML can provide to understand and perhaps control future outbreaks. Needless to say, despite the immense sequencing effort undertaken, our coverage of the world of pathogens remains a drop in the ocean. The lion’s share of SARS-CoV-2 surveillance sequencing is performed by precious few nations and laboratories, making our datasets shortsighted at best. Yet, should the current trends in pathogen sequence data collection continue, perhaps in a future powered by a handful of big-data resources like Global Initiative on Sharing Avian Influenza Data (GISAID),^2^ ML may drive the new era of infection biology and change our approach to emerging pathogens from reactive to proactive.
